# Production and Characterization of Gelatin Biomaterials Based on Agave Microfibers and Bentonite as Reinforcements

**DOI:** 10.3390/foods11111573

**Published:** 2022-05-27

**Authors:** Isidra Guadalupe Ruiz-Martínez, Denis Rodrigue, Martha Lucía Arenas-Ocampo, Brenda Hildeliza Camacho-Díaz, Sandra Victoria Avila-Reyes, Javier Solorza-Feria

**Affiliations:** 1 Centro de Desarrollo de Productos Bióticos, Instituto Politécnico Nacional, calle CEPROBI No. 8, Col. San Isidro, Yautepec C.P. 62731, Morelos, Mexico; iruizm1500@alumno.ipn.mx (I.G.R.-M.); mlarenas@ipn.mx (M.L.A.-O.); bcamacho@ipn.mx (B.H.C.-D.); 2 Department of Chemical Engineering and CERMA, Université Laval, Quebec City, QC G1V 0A6, Canada; 3 CONACyT-Instituto Politécnico Nacional, Centro de Desarrollo de Productos Bióticos, calle CEPROBI No. 8, Col. San Isidro, Yautepec C.P. 62731, Morelos, Mexico; sandra_victory@yahoo.com

**Keywords:** gelatin, agave microfibers, bentonite, biofilms, characterization

## Abstract

The objective of this work was to obtain biomaterials as gelatin films or biofilms produced by casting, reinforced with a microfiber (MF) from *Agave angustifolia* Haw bagasse and bentonite (BN) nanoparticles and evaluate the effect of such reinforcements at different concentrations. Agave microfibers were obtained by a non-abrasive chemical method. Three formulations based on gelatin with glycerol were reinforced with microfiber, bentonite and both materials with 1.5, 3.5 and 5.5% *w*/*w* solids content. Physicochemical properties were determined using SEM and FTIR, thickness, soluble matter and moisture. The XRD, barrier, mechanical and thermal properties were measured. The films’ micrographs showed agglomerations on the surface. Interactions between its functional groups were found. The solubility increased when the MF concentration increased. The thickness of the films was between 60 and 110 μm. The crystallinity ranged from 23 to 86%. The films with both MF and BN and 3.5% *w*/*w* solids had the lowest barrier properties, while the film with 5.5% *w*/*w* solids showed the highest mechanical properties, being thermally resistant. Overall, Agave microfibers together with bentonite were able to improve some of the films’ properties, but optimized mixing conditions had to be used to achieve good particle dispersion within the gelatin matrix to improve its final properties. Such materials might have the potential to be used as food packaging.

## 1. Introduction

Biopolymers are organic substances present in natural resources [1]. Natural polymers are classified into three categories based on their chemical structures [2]: polysaccharides, proteins and polyesters. Common protein-based biopolymers, such as albumin, gelatin and vegetables, have been used in the preparation of nanostructured molecules because of their advantages, such as small size, non-toxicity, long-term stability and biodegradability [3].

Gelatin is a protein derived from the partial hydrolysis of collagen present in the bones and skin of animals. It is a complex polypeptide used in applications such as pharmaceuticals, photography, cosmetics and more widely in the food industry [4]. It is abundant in nature, and its production is not difficult. However, the use of gelatin films in packaging is limited due to its poor mechanical, thermal and mainly water vapor permeability properties due to its hygroscopic nature. This is why several methods have been proposed to improve these properties, such as mixing gelatin with other proteins or polysaccharides, adding crosslinkers, plasticizers and adding micro- and nanoparticles [5].

Agave, also called maguey, belongs to a large botanical family called Agavaceae [6]. Ramírez [7] reported that, of the 273 described species of the entire family, they are all distributed over the American continent from North Dakota (USA) to Bolivia and Paraguay. In addition, García-Mendoza [8] carried out a study of the distribution, endemism and abundance of the different species of the Agavaceae family in Mexico. It was found that, out of a total of 155 species of the Agave genus, 116 (75%) are located in Mexico, of which 90 (58%) are endemic. Commercially, the stem or “piña” of Agave angustifolia Haw is the most important part of the plant, because it is the only part used in the production of alcoholic drinks such as mezcal and bacanora. A by-product derived from the agave stem, generated after the extraction process of its commercial products, is bagasse, which is composed of residual fiber and represents 40% of the processed plant [9]. The annual generation of bagasse in Mexico is approximately 1.05 × 108 kg, representing usually 40% in dry weight of the processed agave [10]. This particular situation is very attractive to investigate for a better use of agave bagasse, which is composed of cellulose microfibrils surrounded and packed with hemicellulose and lignin among several other components [11], forming a lignocellulosic matrix, which has generated great interest in the scientific community for its use and waste mitigation.

Nanoclays are a type of layered silicate, and bentonite is one of the most popular and abundant examples. Bentonite has been commonly used in several applications such as water purification, clarification and purification of sugar solutions, sewage and effluent treatment, pharmaceutical and therapeutic preparations, carrier for catalysts, refining and bleaching of glyceride oils carrier for fungicides, drilling fluids, fertilizer sprays, wall support for boreholes, formulation of ceramic glazes, non-drip paints, formulation of mortars, putties, adhesives, pelletizing animal feedstuffs, building agents for special molding sands and reinforcement of polymeric materials [12]. This type of nanoclay has a high ion exchange capacity and a negative surface charge. It is composed of a stack of silicate sheets dispersed in individual layers, separated by voids in which exchangeable cations are present. The nanoparticles have an ultrafine phase dimension, typically in the range of 1–100 nm, and strong interfacial interactions between the dispersed clay layers and polymeric matrices [13].

Although the casting method for obtaining biofilms, it is not particularly a highly technological method used in the traditional packaging industry, but it is easy, inexpensive and possible to be performed anywhere under optimized conditions. So, it is a good starting point to give an overview of the appropriate formulations of filmogenic solutions, drying conditions, physical or chemical interactions between the components and to determine the feasibility of biofilms production. From this, it provides knowledge about the biopolymers and the importance of using natural reinforcements, thus reporting the viability of the biomaterials obtained. Then, the results can be transferred to industrial scales, probably benefiting the environment and the people, encouraging the use of biodegradable materials.

In this work, formulations from gelatin with different concentrations of reinforcements (agave microfiber and bentonite) are proposed, which are able to produce filmogenic solutions and subsequently develop biofilms by the casting method. The characterization of their chemical, mechanical, morphological, physical and thermal properties is also presented to evaluate the effect of the reinforcements (alone or together) on gelatin biofilms.

## 2. Materials and Methods

### 2.1. Materials

The materials used for this study were: gelatin (Berthelet, Laval, QC, Canada), glycerol (GLY) (analytical grade, Sigma-Aldrich, Saint Louis, MO, USA), hydrophilic bentonite (Nanomer^®^ PGV, Sigma-Aldrich, Saint Louis, MO, USA), lignocellulosic microfiber from *Agave angustifolia* Haw, obtained from the method developed by Hernández et al. [14], potassium hydroxide, hydrochloric acid and silica gel (Hycel, Jalisco, Mexico), sodium bromide and sodium chloride (Fermont, Monterrey, Mexico). Distilled water (DW) was used in all formulations.

### 2.2. Biofilm Formulations

The biofilms, named from F1 to F10, were prepared using the casting method (Table 1), with gelatin as the base macromolecule of the polymeric matrix, plasticized with glycerol and water, and reinforced with different concentrations of agave microfiber and nanoclay (bentonite). All the film-generating solutions or filmogenic solutions were made by fixing the concentration at 6% *w*/*w* of total solids.

### 2.3. Methods

#### 2.3.1. Filmogenic Solutions

To produce the filmogenic solutions for each biofilm (F1 to F10), the methodology of Bae et al. [15] and Mondragon et al. [16] were followed, with some modifications. Firstly, aqueous suspensions of gelatin and agave microfiber were prepared. Then, the necessary weight of gelatin (GL) was left to hydrate with about 23.5 mL of water (DW) for 3 h at room temperature. Then, the sample was kept under agitation for 20 min at 50 °C and the pH was adjusted to 9.0 with a 1 M KOH solution. Furthermore, the corresponding amount of agave microfiber (MF) was added in 23.5 mL of DW and stirred at room temperature for 2 h to get a suspension which was immersed in an ultrasonic bath for 30 min at 60 °C using a Branson brand sonicator (Model 2510, Danbury, CO, USA) (40 kHz, 75 W). Finally, the GL solution was added to the agave microfiber (MF) suspension, and this mixture was sonicated (40 kHz) for 25 min before being stored at room temperature.

The methodology to produce filmogenic solutions and biofilms was based on the work of Bae et al. [15], with some modifications for bentonite and glycerol solutions as mentioned below. Glycerol (GLY) was dissolved in 47 mL of DW, followed by stirring for 30 min at 50 °C. Then, the amount of BN for 100 g according to the selected formulation was slowly added and stirred again for 30 min at 50 °C. The suspension obtained was homogenized at 4500 g for 10 min (ULTRA-TURRAX IKA, T25 digital, Deutschland, Germany). Subsequently, the GLY-BN solution was gently added to the GL-MF solution by dripping and constant stirring for 6 h at 50 °C. Then, both mixtures (GL-MF and GLY-BN) were homogenized at 4500 g for 10 min, followed by sonication (40 kHz) for 30 min, in order to achieve good exfoliation and intercalation of the nanoclay.

#### 2.3.2. Biofilm Elaboration by the Casting Method

The biofilms (F1 to F10) were prepared using the casting method [15,16,17]. The filmogenic solutions were cast onto square acrylic boxes (Corning) measuring 24.5 cm × 24.5 cm, which were dried at 35 °C for 24 h. Finally, the biofilms were removed and stored for 7 days at room temperature in a desiccator, with a relative humidity of 57%, conditioned by a saturated solution of sodium bromide (NaBr).

#### 2.3.3. Surface and Cross-Section Characterization of the Biofilms by SEM

All the biofilms prepared (Table 1) were analyzed with a scanning electron microscope (EVO LS10, Carl Zeiss, Promenade, Germany), which was operated in the environmental mode at 20 kV. Cryogenic fractures of films were made with liquid nitrogen. Images were taken at different magnifications (200× and 500×) using a backscattered electron detector. Thus, micrographs of the surface and cross-section of each biofilm were taken.

#### 2.3.4. Detection and Analysis of Functional Groups in the Biofilms by FTIR

The biofilms were preconditioned for 7 days in a desiccator with silica gel, reaching a proximate absence of moisture (close to 0%). All samples were analyzed in triplicate (*n* = 3) with a FTIR spectrophotometer Shimadzu TM (IR Affinity model, Shimadzu, Kyoto, Japan), equipped with ATR (attenuated total reflection). The spectra were determined in the absorbance mode, region used for the analysis was in the range from 600 to 4000 cm^−1^, number scans: 30, 4 cm^−1^ resolution, (at 40% RH).

#### 2.3.5. Determination of the Soluble Matter

The solubility content of the biofilms was determined according to the modified methodology proposed by Cuq et al. [18]. Each sample was cut square (2 cm × 2 cm) and dried in an oven at 40 °C for 24 h, to reach a constant initial weight ($W_{i}$). Then, each sample was immersed in 30 mL of distilled water for 24 h at room temperature. Subsequently, the water was removed by decantation and the biofilms were dried again at 40 °C for 24 h to obtain their final dry weight ($W_{f}$). The solubility percent (% SP) was determined with the following equation:

(1) $$\%\;SP\;=\;\left(\frac{W_{i}\;-\;W_{f}}{W_{i}}\right)\;\times \;100$$

The average value was calculated based on three replicates for each biofilm.

#### 2.3.6. Determination of Moisture Content

The moisture content of the biofilms was determined in triplicate according to the methodology reported by Rhim et al. [19]. The method involves measuring the weight loss of the biofilms when dried in an oven at 105 ± 3 °C for 24 h. The moisture content was calculated as:

(2) $$\%\;\mathrm{Moisture}\;\mathrm{content}\;=\;\left(\frac{M_{w}\;-\;M_{d}}{M_{w}}\right)\;\times \;100$$

where $M_{w}$ is the weight of the biofilms conditioned at 65% RH to moisture equilibrium and $M_{d}$ is the dry weight of the biofilms.

#### 2.3.7. X-ray Diffraction

Diffraction patterns were recorded using an X-ray diffractometer Miniflex 600 (Rigaku, Tokyo, Japan) equipped with a Cu Kα radiation source (λ = 0.154 nm) operating at 45 kV and 15 mA. The scanning speed was 3 deg min^−1^ with a scanning step of 0.01° at 25 °C. The diffracted intensity was measured from 3° to 60° in 2θ.

#### 2.3.8. Barrier Properties NY

Water vapor transmission tests were performed according to ASTM E96-93 (1993), also known as the cup or the test cell method. Before testing, the biofilms were conditioned in a desiccator (RH close to 57% supplied by saturated sodium bromide) for five days at 25 °C. Three replicates (*n* = 3) were tested for each sample. Then, the biofilms were fixed on the top of the test cups containing a desiccant (silica gel). The test cups were placed in an environmental chamber at a controlled temperature (25 °C), and a saturated solution of NaCl was placed into the desiccator (75% RH). The mass change (±0.0001 g) of the cups as a function of time was recorded at specific time intervals (t). After the permeation analysis, the biofilm’s thickness was measured and the water vapor permeability, *WVP* (kg m s^−1^ m^−2^ Pa) was calculated using Equation (3).

(3) $$WVP=\left[\frac{G\cdot x}{t\cdot Ae\cdot S\cdot \left(R1-R2\right)}\right]$$

where:

x = biofilms mean thickness (m), Ae = exposed area (m^2^), S = water’s saturated vapor pressure at the test temperature (Pa), R1 = dessicator RH, R2 = permeation cell RH, G/t (kg/s) is the linear regression angular coefficient of the system vs. time weight gain straight-line equation. All the measurements were done in triplicate (*n* = 3) for each biofilm.

#### 2.3.9. Mechanical Properties

The mechanical properties of the different biofilms were determined by testing the tensile strength at fracture (TS), strain at break (SB) and elastic modulus (EM) according to ASTM D882-02 (2002). Firstly, the samples were conditioned for 7 days in a desiccator with a saturated NaBr solution providing 57% RH. The tests were performed in a TAXT2i Texturometer (Stable Micro Systems^TM^, Surrey, UK) equipped with a 25 kg load cell with an effective clamp spacing of 60 mm and a strain rate of 10 mm/min. Rectangular samples (1 cm × 10 cm) were cut in the biofilm and the thickness was determined by a Mitutoyo IP65 digital micrometer (Coolant Proof, Kawasaki, Kangawa, Japan) at 10 random positions. The TS, SB and EM values were obtained directly from the force as a function of elongation data using the computer software. The tests were performed ten times (*n* = 10) for each formulation.

#### 2.3.10. Thermogravimetric Analysis

A thermogravimetric analyzer (DISCOVERY TGA-5500, TA Instruments, New Castle, DE, USA) was used and connected to a computer for control and data analysis via the software Trios (V4.5.42498). The tests were performed according to the method suggested in the literature ASTM D882-02 (2002). A platinum pan was filled with 2–8 mg of sample. The measurement was done from 45 °C up to 800 °C at a heating rate of 10 °C min^−1^. Pure nitrogen gas was circulated at a rate of 20 mL min^−1^ through the oven to maintain an inert atmosphere. The first derivative of the thermogravimetric curve as a function of temperature was also evaluated. All the measurements were taken in triplicate (*n* = 3).

#### 2.3.11. Statistical Analysis

Statistical analyses were performed using OriginPro 8 (OriginLab Corporation, Northampton, MA, USA). The data were subjected to an analysis of variance to determine significant differences between the samples (*p* < 0.05). If significant differences were detected, the average values were compared using a Tukey’s test and differences were considered significant when *p* < 0.05.

## 3. Results and Discussion

### 3.1. Surface and Cross-Sectional Characterization

**Figure 1:**
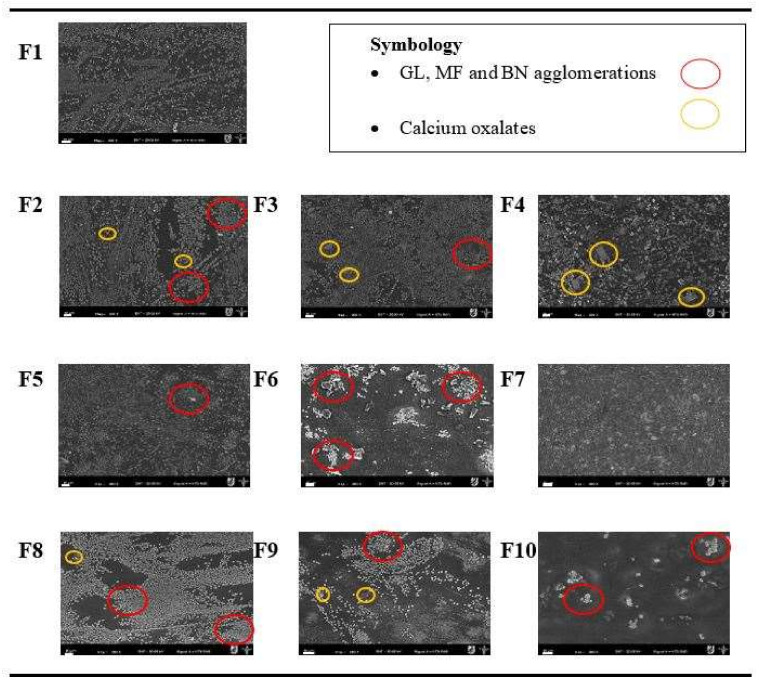
Superficial micrographs obtained by scanning electron microscopy (SEM) of the reinforced gelatin biofilms. The scale bar indicates 20 µm, SEM magnification: 200×. F1: control sample; F2–F4: samples with microfiber; F5–F7: samples with bentonite; F8–F10: samples with microfiber and bentonite. MF = Agave microfiber; BN = Bentonite; GL = Gelatin; GLY = Glycerol; DW = Water.

The morphology of the surface of all biofilms produced by casting is shown in Figure 1 as obtained by scanning electron microscopy (SEM). In most of the biofilms, a regular continuous surface was observed without gaps, voids, or cracks as reported by Fakhouri et al. [20]. However, there were some agglomerations of the components as indicated by the red circles, which could induce defects in the materials and affect their mechanical and barrier properties. These defects could be linked to the hydrophobic character of the MF, due to the lignocellulosic content and the limited dispersion of BN.

On the surface of the control biofilm (F1), some heterogeneous dispersion was observed with respect to the alignment of insoluble gelatin molecules. For biofilms F2 to F4, in addition to some insoluble gelatin, agave microfibers were observed distributed over the surface which were more noticeable as their concentration increased. Some probable MF impurities can be observed, mainly calcium oxalate crystals in the form of prisms inserted in the protein matrix as indicated in Figure 1 (yellow circles). Monje et al. [21] also reported that plants of the Agavaceae family can synthesize different chemical forms of calcium oxalate. In biofilms F5 and F7, a dispersion of the microstructures was observed, which could be attributed to an adequate sonication during bentonite addition, while in F6, components agglomeration is observed. The literature reports that bentonite behaves like polydisperse particles, which cannot cross-link with the protein matrix due to agglomeration, leading to their presence on the biofilm surface [17].

**Figure 2:**
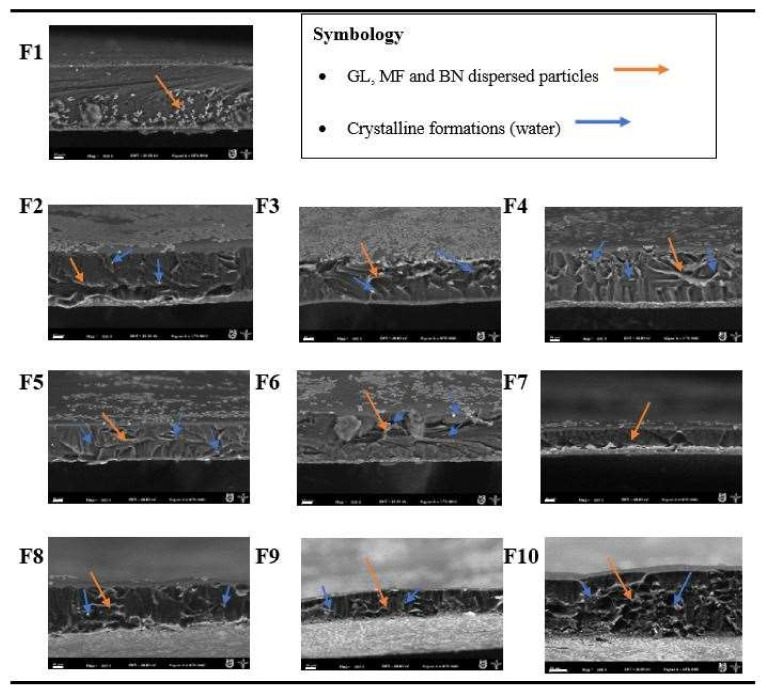
Cross-sectional micrographs obtained by scanning electron microscopy (SEM) of the reinforced gelatin biofilms. The scale bar indicates 10 µm, SEM magnification: 500×. F1: control sample; F2–F4: samples with microfiber; F5–F7: samples with bentonite; F8–F10: samples with microfiber and bentonite. MF = Agave microfiber; BN = Bentonite; GL = Gelatin; GLY = Glycerol; DW = Water.

A combination of all the aforementioned phenomena occurred in biofilms F8 and F9, where it was observed that with increasing MF and BN concentrations, agglomerations on the surfaces were more evident due to inadequate dispersion and compatibility between the components. In turn, sample F10 showed on its surface more packed agglomerations or clumps of its components. This is why it was not possible to visualize the presence of calcium oxalates, but these should not be ruled out. In the cross-section of all biofilms (Figure 2), smooth, thin, void-free and crack-free micrographs were obtained, an indication that a more compact internal structure of the samples was achieved.

The biofilms were immersed in liquid nitrogen to expose the cross-section, which might cause some crystalline formations or possibly water fragments, indicating that each gelatin matrix being plasticized with glycerol and water had a high content of free and bound water able to form this type of structure in the protein biofilms, as also reported by Ortiz-Zarama et al. [17] in their plasticized protein biofilms. The thickness of the biofilms decreased (see later) and became more compacted, due to the addition of the reinforcements used (MF and BN). In addition, a random dispersion and agglomeration of the reinforcements on each sample was observed.

### 3.2. Detection and Analysis of Functional Groups

**Figure 3:**
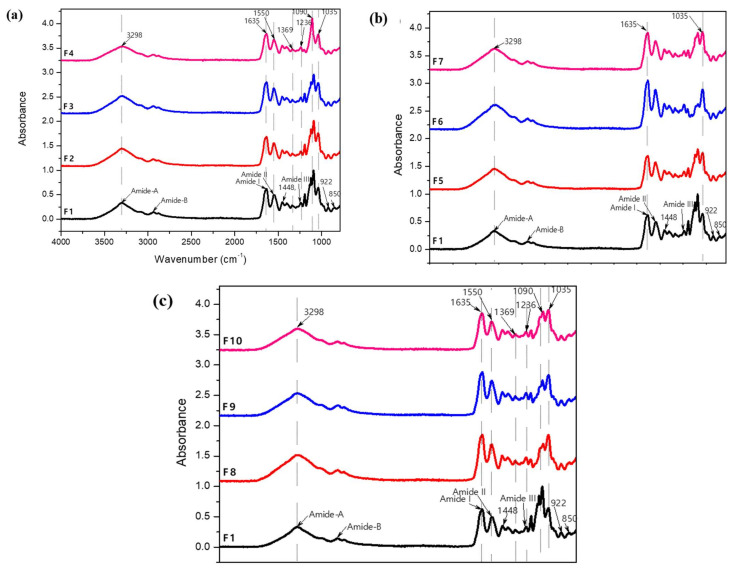
Fourier Transform Infrared Spectroscopy (FTIR) results of the reinforced gelatin biofilms. (**a**) Spectra of biofilms F1 to F4, (**b**) spectra of biofilms F1, F5 to F7 and (**c**) spectra of biofilms F1, F8 to F10.

Figure 3 presents the FTIR spectra of gelatin biofilms reinforced at different MF and BN concentrations compared with the control biofilm (F1). In addition, the wavenumbers, vibrations, functional groups and corresponding biofilms are listed in Table 2.

In Figure 3a, the spectra of biofilms F2, F3 and F4 compared to the control biofilm (F1) show the presence of the lignocellulosic microfibril by the bands at 3298 cm^−1^ (due to intermolecular OH hydrogen bonds of cellulose), 1448 cm^−1^ due to asymmetric CH deformation in methoxyl groups -OCH_3_ present in lignin. In general, all the spectra had the same behavior with respect to the polymeric matrix (gelatin), but with notable differences.

All infrared spectra showed the main vibrational maxima (peaks) characteristic of the proteins: 3305–3280 cm^−1^ and 2994–2904 cm^−1^ (corresponding to amide-A and amide-B, respectively, illustrating NH stretching coupled with hydrogen bonding), the band at 1635 cm^−1^ is related to amide-I, illustrating C = O stretching vibration coupled with in-plane bending of NH bond and CN bond stretching. The signal at 1550 cm^−1^ is the amide-II, representing NH bending and CN stretching. The CH deformation at 1448 cm^−1^ and 1236 cm^−1^ are attributed to amide-III, associated with in-plane vibration of the CN and NH groups of the bonded amine together with oscillation vibrations of the CH_2_ groups of the glycine skeleton and proline side chains [22,23,24]. Additional peaks were observed at 1035 cm^−1^, 922 cm^−1^ and 850 cm^−1^, which represent the interaction between the plasticizer (OH groups of glycerol) and the gelatin biofilm structure [25,26].

In Figure 3b, the presence of bentonite in its different concentrations in samples F5, F6 and F7, compared with the control biofilm (F1), was verified. The broad bands at 3298 and 1635 cm^−1^ represent the stretching and bending vibrations of the water molecules by the OH (hydroxyl), and then, intensities of the bands were increased, suggesting that the different concentrations of BN caused conformational changes in the secondary structure of the proteins [27,28].

This effect is probably due to interactions of the silica groups of BN with the carboxylic groups of gelatin, which generate hydrogen bonds and thus change the IR absorption of the COO-groups. Caccamo et al. [29] identified the spectral characteristics of the groups associated with octahedral cations, quartz, silicates and water present in bentonite. This is consistent with this work where the bands at 1035 cm^−1^ (stretching in the Si-O plane) and at 922 cm^−1^, for the bending vibration of the OH group coordinated to the cations, established for Al-Al-OH as mentioned by Malhotra et al. [30] and Vargas-Rodríguez et al. [31].

Figure 3c shows the spectra of F8, F9 and F10 compared to the control biofilm (F1), the presence of all the mixed components (GL, GLY, WT, MF and BN) of each biofilm was verified to confirm the presence of the most representative functional groups, where the peaks had intermediate absorbance values compared to those obtained in Figure 3a,b. The components, especially in the amide-A bands, N-H stretching associated with the protein matrix, coupled with the hydroxyl (-OH) bonds of the plasticizers, were observed. In addition, the peaks at 1090 cm^−1^ and 1035 cm^−1^ showed the interaction between the pectin structures (C-O bending) and the silica tetrahedron (stretching in the Si-O plane).

### 3.3. Determination of Soluble Matter in Aqueous Solution and Moisture Content

**Figure 4:**
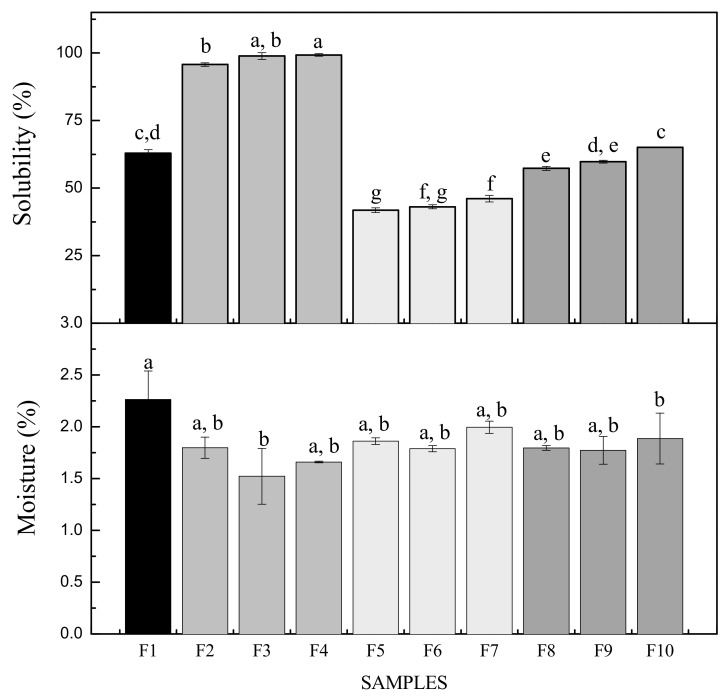
Solubility and moisture content of the reinforced gelatin biofilms. Different letters indicate statistically significant differences (*p* < 0.05) among the biofilms (*n* = 3). F1: control sample; F2–F4: samples with microfiber; F5–F7: samples with bentonite; F8–F10: samples with microfiber and bentonite.

The water solubility values of the control and MF and BN reinforced gelatin biofilms are shown in Figure 4. Solubility is an important parameter to control the ability of a biofilm to maintain its structural integrity in an aqueous environment. The solubility for the control biofilm (F1) was around 63%, which is consistent with the works of Jiang et al. [32] and Hosseini et al. [33]. This high value is attributed to the hydrophilic nature of gelatin (presence of polar peptides in gelatin) combined with the hydrophilic plasticizer (glycerol) added to F1 to provide adequate flexibility to the biofilms.

In biofilms F2 to F4, the values increased and with significant differences (*p* < 0.05) with respect to F1: the solubility increased steadily from 95 to 98% when the MF concentration increased (Figure 4). Jiang et al. [32] and Pérez-Mateos et al. [34] found similar results with gelatin biofilms reinforced with different particles such as triacetin, sunflower oil, stearic and palmitic acids. This solubility increase was attributed to the limited formation of intermolecular bonds between the matrix components and the reinforcements, being consistent with the results obtained in the FTIR spectra (Figure 3). The hydrophobic nature of the microfiber due to its lignocellulosic composition made it insoluble to water. In contrast, the solubility in samples F5 to F7 decreased and presented significant differences (*p* < 0.05) with respect to F1, being in a range from 41 to 46%. This decrease in solubility is due to the formation of hydrogen bonds with the GL and BN as observed in the FTIR spectra (Figure 3). The biofilms with both reinforcements (MF and BN) had similar solubility values in F9 and F10, since they did not present significant differences (*p* > 0.05). This result is attributed to the operating conditions, especially to the sonication of the filmogenic solutions, allowing the interaction between the matrix and the bentonite, in addition to the dispersion of the microfibers. In the case of sample F8, a decrease in the solubility percentage was observed and there was a significant difference (*p* < 0.05) with respect to F1, it behaved as the ideal formulation when the reinforcements (MF and BN) were added, due to the fact that there were physical interactions between its components as was verified on the superficial micrographs in Figure 1 and observed in the spectra (FTIR) in Figure 3c. It was also reflected in the water vapor permeability results, being the best biofilm (F8) showing the lowest value in that property.

The moisture content of biofilms is very important for food packaging applications. The moisture content for all the reinforced biofilms (F2–F10) did not have a significant variation (*p* > 0.05) compared to the control biofilm (F1) as observed in Figure 4. The range of moisture values in this work is between 1.6 and 2.2%, while previous works reported higher variation among their moisture values. For example, biofilms based on gelatin from different sources and using the casting method led to moisture contents of 5.2–6.7% [22], 14–21% [35] and 20–40% [36]. These latter authors reported that the presence of glycerol allowed the absorption of more moisture due to its hydrophilic nature, which is consistent with our results, without significant differences in the moisture content for all the biofilms obtained in the present work, since the glycerol concentrations in each sample were similar. The intermolecular interactions present in each sample bound by their compatible functional groups, such as hydrons, carbonyls, amides and sulfates, built a stable cross-linked network unable to absorb moisture from the external environment.

### 3.4. Thickness

The thickness of the control biofilm (F1) and those reinforced with MF and BN at various concentrations are also presented in Table 3. In general, the thickness of a biofilm depends on the composition of the biofilm-forming solution, as well as the nature and concentration of its components. So, the components of a biofilm forming solution affect the alignment, arrangement and compaction of molecules during the biofilm drying process, which causes the differences in thickness [37,38]. The thickness of the F1 biofilm showed the highest value, due to the absence of the reinforcements and the higher concentration of GL and GLY, probably glycerol molecules penetrate rapidly in the gelatin network to contribute to the formation of a thicker biofilm.

The stability of this compound depends on the length of the polymer chain (gelatin), its cross-link density and the amount of adsorbed water [39]. Wu et al. [40] reported that gelatin molecules available within a polymeric matrix form a unique network structure via hydrogen bonds, and upon contact with glycerol increase the network structure. As the concentrations of gelatin and glycerol decreased for each sample prepared, besides the addition of the reinforcements, the thickness of the biofilms significantly decreased (*p* < 0.05). The thicknesses obtained in this work were within the range of 60.0 to 110 μm. The thickness of commercial plastic films ranges usually from 120 to 125 μm for polypropylene (PP), while for low-density polyethylene (LDPE) the thickness lies within a range of 15–250 μm [24]. This suggests that all the biofilms produced could be acceptable for commercialization (Table 3).

### 3.5. Crystallinity and Amorphous Matter

**Figure 5:**
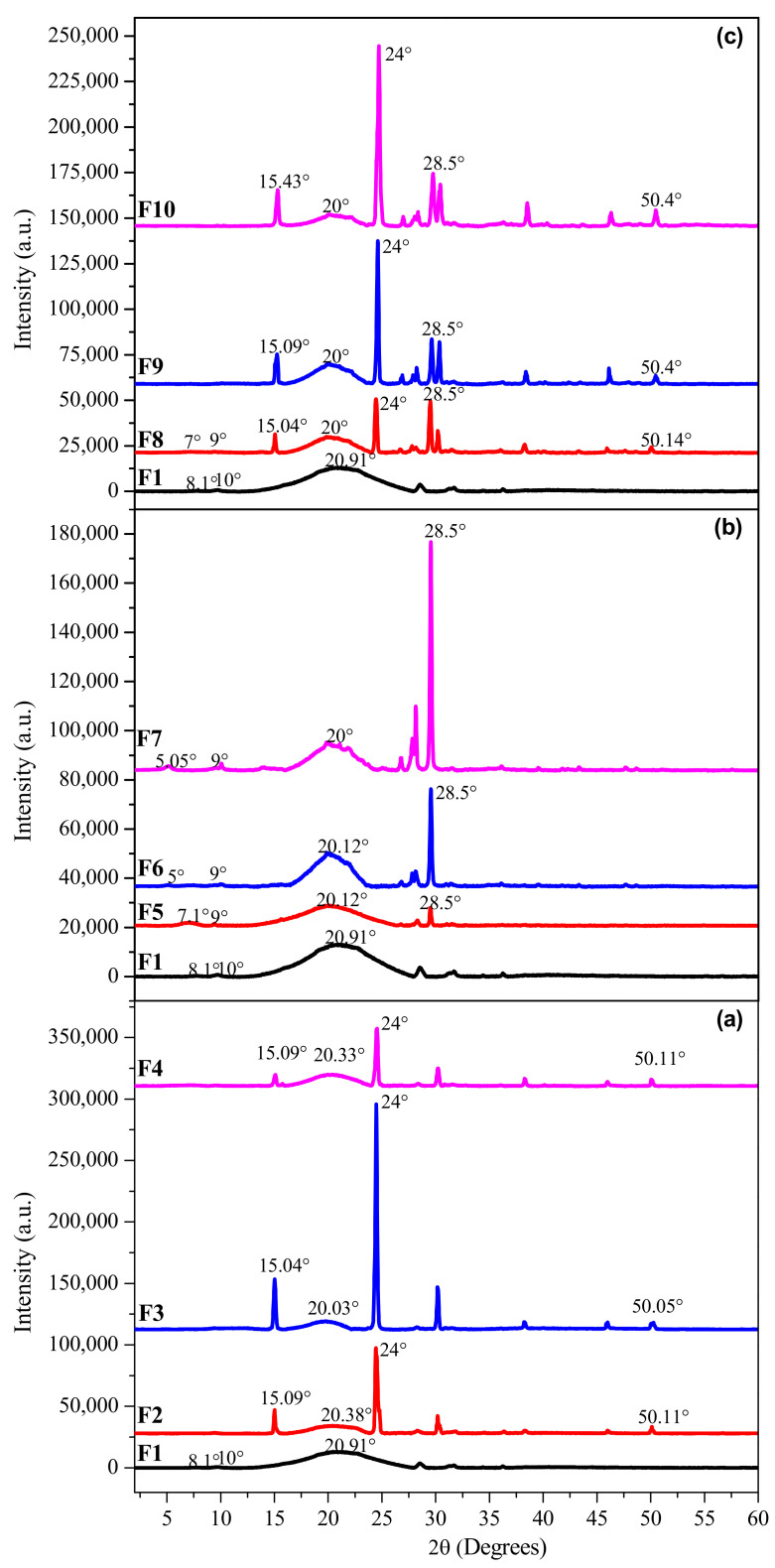
X-ray diffraction patterns of the reinforced gelatin biofilms. (**a**) Diffractograms of biofilms F1 to F4, (**b**) Diffractograms of biofilms F1, F5 to F7 and (**c**) Diffractograms of biofilms F1, F8 to F10.

X-ray diffraction (XRD) patterns are presented in Figure 5 to compare the gelatin biofilms reinforced with MF and BN (F2–F10) with the control biofilm (F1). F1 shows two weak diffraction peaks around 2θ = 8.1° and 10°. These peaks indicate the reconstitution of the collagen triple helix structure. In addition, a broad profile was found with the main diffraction of the amorphous peak at 2θ = 20.9° related to the distance between amino acid residues along the helix, which is about 0.44 nm [41]. Reflections at 2θ = 20° and 50° are characteristics of gelatin, confirming the presence of the amorphous matrix and the reconstitution of the triple helix structure of collagen. In Figure 5a, the absence of the first peaks in the control biofilm (2θ = 8.1° and 10°) was observed, but new peaks characteristic of a lignocellulosic material such as MF were also detected around 2θ = 15° and 24°, which correspond to fibers from *Agave angustifolia* Haw [42], which is associated with the presence of residual oxidized lignin or pectin characteristics of cellulose (I). In Figure 5b, new diffraction peaks were presented around 2θ = 7° and 9° for F5. For F6 and F7, a peak was present at 2θ = 5° and a constant peak at 2θ = 28.5° for F5–F7. Figure 5c, showed the presence of all the characteristic peaks of GL, MF and BN together, the intensities of the peaks decreased due to the interferences of the reinforcements dispersed in the protein matrix.

The crystallinity index was calculated from the formula of Nam et al. [43] and Kazachenko et al. [44], giving 0.19 for F1. The crystallinity index decreased in the order F2 > F3 > F4 which corresponds to 0.76 > 0.72 > 0.65, respectively, due to higher microfiber concentration. These values were similar to those reported by El Oudiani et al. [45] and Flores-Sahagun et al. [46] who evaluated different types of agaves in Mexico. A decrease in the crystallinity was observed for F5 (0.69) > F6 (0.53) > F7 (0.23), due to higher bentonite concentration and also for F8 (0.86) > F9 (0.75) > F10 (0.66), due to the synergy between MF and BN. The biofilms with MF had a higher crystallinity compared to biofilms filled with BN, the latter improved the physicochemical properties thanks to electrostatic and chemical interactions with the gelatin matrix, making it more resistant as described via mechanical and thermal properties.

### 3.6. Barrier Properties

**Figure 6:**
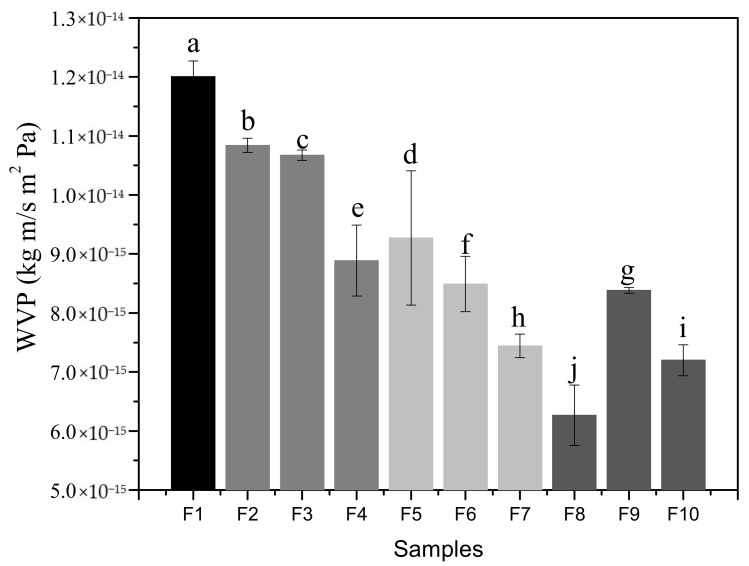
Barrier properties of the reinforced gelatin biofilms. Different letters indicate statistically significant differences (*p* < 0.05) between the biofilms (*n* = 3). F1: control sample; F2–F4: samples with microfiber; F5–F7: samples with bentonite; F8–F10: samples with microfiber and bentonite.

The values of water vapor permeability (WVP) are shown in Figure 6. Since one of the main functions of a food package is usually to prevent or at least decrease moisture transfer between the food and the surrounding atmosphere, or between two components of a heterogeneous food product, the WVP should be as low as possible [47]. The results in Figure 6 for F1 showed the highest value of WVP, having significant differences (*p* < 0.05) compared to the other biofilms (F2 to F10). Apparently, the thickness of the biofilms did not influence the water permeability results. It was shown that reinforcing the biofilms with MF and BN led to significant changes between the samples when the water molecules passed through the biofilms over a certain time. Overall, the results decreased when the concentrations of MF and BN increased.

The control biofilm (F1) showed the highest value because it also had the greatest thickness (106 µm), this allowed more water to be absorbed from the environment since the gelatin used as the matrix is hydrophilic, providing the ability to bind water molecules by means of hydrogen bridges [48]. Additionally, the character of the plasticizer itself is another important factor to take into account in F1 elaborated with 30% *w*/*w*, having the highest concentration of glycerol of all samples. This allowed the water content in the biofilm to increase, consequently increasing chain mobility or flexibility as observed in the mechanical properties. Núñez-Flores et al. [25], Núñez-Flores et al. [49] and Nor et al. [50] obtained WVP results similar to those obtained for F1 (7.22 × 10^−14^ kg m/s Pa m^2^, 5.72 × 10^−14^ kg m/s Pa m^2^ and 6.67 × 10^−14^ kg m/s Pa m^2^, respectively), who prepared gelatin biofilms plasticized with glycerol as a control sample. The addition of micro and nanoparticles to the gelatin matrix at different concentrations had an effect on the WVP results. The samples reinforced with MF sole (F2 to F4) gave WVP values between 1.08 × 10^−14^ and 8.89 × 10^−15^ kg m/s Pa m^2^, being even higher and significantly different to the samples reinforced with BN sole (9.27 × 10^−15^–7.44 × 10^−15^). These lower water vapor permeabilities can be related to the reduction of free volume and a more compact structure due to the presence of these reinforcements, where they did not allow the passage of water molecules in the environment. Similarly, the presence of intercalated and exfoliated structures of the clay created more tortuous paths. Hong et al. [51] studied the mechanical properties of chitosan biofilms reinforced with nanocomposites, reporting a decrease in WVP with increasing reinforcement concentration, due to the negatively charged clay acting as an ionic crosslinker and its ability to adsorb water. Samples reinforced with both MF and BN components resulted in the lowest WVP values, especially F8 (1.5% *w*/*w* of each reinforcement) leading to 6.27 × 10^−15^ kg m/s Pa m^2^. The thickness of each sample depends on the compaction of the reinforcements during drying, F8 has a highly compacted structure, as observed in the micrographs of the biofilm cross-section (Figure 2), a better dispersion of the reinforcements more effectively prevented the penetration of water vapor. However, there was a variation in samples F9 and F10, which continued to manifest low values with respect to the other samples, probably this resulted in the development of a heterogeneous biofilm structure, due to the excess of micro and nanoparticles, with discontinuities or irregularities that coincide with the results of the tensile strength and elastic modulus (shown below), which had the same variation and behavior. Biofilms F8, F9 and F10 could be proposed as an alternative to be used in commercial packaging, since their WVP values are close to synthetic polymers, especially cellophane (8.41 × 10^−14^ kg m/s Pa m^2^) and low-density polyethylene (LDPE: 3.6–9.7 × 10^−16^ kg m/s Pa m^2^) as reported by Shellhammer et al. [52].

### 3.7. Mechanical Properties

**Figure 7:**
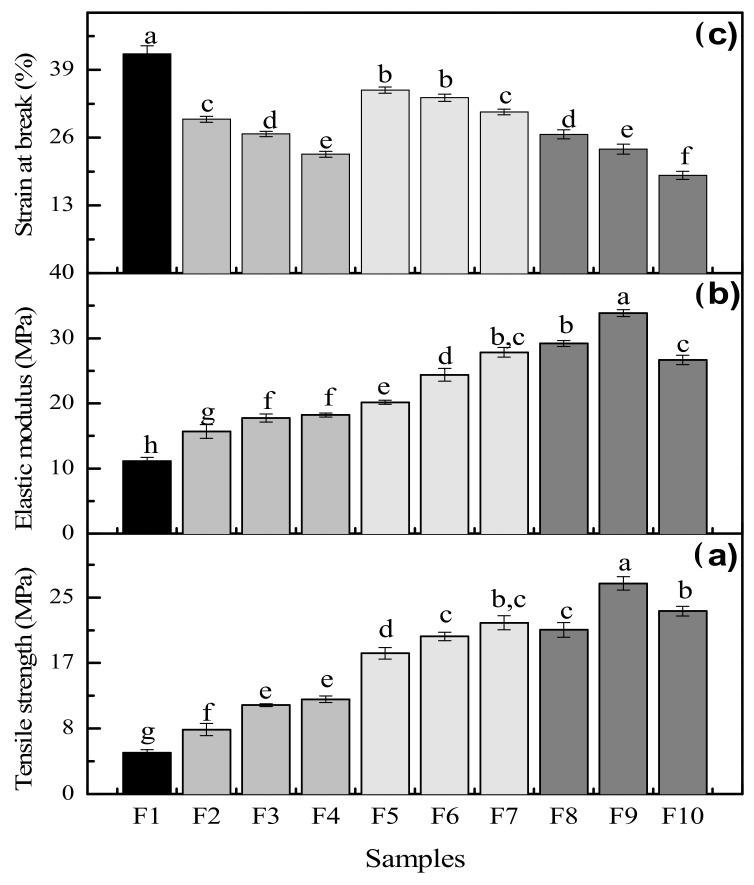
Mechanical properties of the reinforced gelatin biofilms. Average value ± standard deviation (*n* = 10). Values with the same letter did not show significant differences among the samples (*p* > 0.05). (**a**) Tensile strength, (**b**) elastic modulus and (**c**) strain at break of biofilms F1 to F10.

Figure 7 shows the tensile strength at fracture (TS), strain or elongation at break (SB) and elastic modulus (EM) of the biofilms. The control sample (F1) was evaluated to compare with the reinforced samples (F2 to F10). The mechanical properties of biofilms, in general, are largely associated with the nature and chemical structure of the materials making them [53]. Figure 7a clearly shows that the tensile strength of gelatin biofilms significantly increased with increasing micro and nano-reinforcement content. The samples reinforced with MF alone at different concentrations (F2 to F4) presented significant differences (*p* < 0.05) with respect to the control biofilm F1, with values in the range of 8 to 12 MPa, i.e., increases between 157 and 230% when adding the microfiber compared to the control biofilm. Ludvik et al. [54] observed the same behavior in their samples reinforced with cellulosic fiber and the TS, whose values were similar to those obtained in this study, which was due to the firmness and stability of the structure of the lignocellulosic material, providing strength to the biofilms.

The samples reinforced with BN alone also showed significant differences (*p* < 0.5) with respect to the other samples: control sample (F1) and reinforced with MF (F2 to F4), followed the same trend of increasing TS as the nanoclay concentration increased, leading to values from 17 to 21 MPa or a substantial increase between 342 and 416% with respect to F1. The work of Gabr et al. [55] showed the same TS increase with increasing nanocellulose concentration in nanoclay biocomposites. Solorza-Feria et al. [56] reported in their studies that nanoclay intercalates in the gelatin structure, increasing the strength of the reinforced matrix. Thus, of the three biofilms (F8 to F10) with both reinforcements (MF and BN) at their respective concentrations, sample F9 produced the maximum tensile strength (26.7 MPa), increasing substantially with respect to the F1 value (5.21 MPa) by 512%. However, a decrease in sample F10 was observed.

This might be due to a high agglomeration between the micro and nano reinforcements that were not well dispersed in the gelatin matrix, without contributing to reinforcement, in addition to the lower exfoliation level of the nanoclay [55]. Figure 7b shows that the elastic modulus has a similar behavior compared to the tensile strength: EM is increasing with increasing particle concentration up to sample F9. This latter has a 304% increase with respect to the control biofilm F1 with 33.8 MPa. Figure 7c shows that the strain at break of the control F1 is the highest at 42% because it contains the highest concentration of GL and GLY.

Loo et al. [57] obtained a value of 45% for their gelatin biofilm plasticized with glycerol at 30% *w*/*w*. It is known that glycerol is the component giving flexibility and softness to the protein biofilms and to cross-linking bonds making intermolecular bonds stronger, as well as forming hydrogen and carbon-carbon bridges as observed in the FTIR spectra (Figure 3). Samples F2 to F4 showed significant differences (*p* < 0.5) compared to F1 with SB decreasing with increasing MF concentration, and the GLY concentration slightly decreasing.

Since the lignocellulosic material is hydrophobic, a stronger but less elastic material is obtained. The same phenomenon occurs for samples F5 to F7 as the SB decreased with increasing BN concentration. This could be associated with the slight decrease of GLY content and the reduction of BN dispersion quality or exfoliation, leading to the formation of tactoids (agglomerates) of nanoclays [58]. These agglomerations were observed in Figure 1, possibly due to some limitations in the sonication of BN which did not allow a homogeneous dispersion of nanoparticles in the gelatin matrix. Samples F7 to F10 presented the lowest strain at break with values in the 18–26% range, which were significantly different (*p* < 0.5) compared to all other samples and especially with F1. There were some interactions between the individual effect of each reinforcement by combining the micro and nanoparticles (hybrid system), resulting in stronger and more resistant materials, but with a lower elasticity. This behavior confirmed that the selected particles have the ability to improve the TS and EM, which could be optimized depending on the properties of the gelatin matrix, i.e., the MF/BN ratio could be optimized depending on the total reinforcement content. Higher tensile strength and elastic modulus with MF addition are related to the interactions between functional groups with the protein matrix, as observed in the FTIR spectra (Figure 3), forming hydrogen bridges leading to higher resistance of these biofilms. In addition, a higher crystalline material is obtained due to the presence of microfiber. The dispersion of bentonite in the matrix form electrostatic bonds between the clay layers, while hydrogen bridges and covalent bonds generate higher resistance to the materials, although involving amorphous materials.

### 3.8. Thermogravimetric Analysis

**Figure 8:**
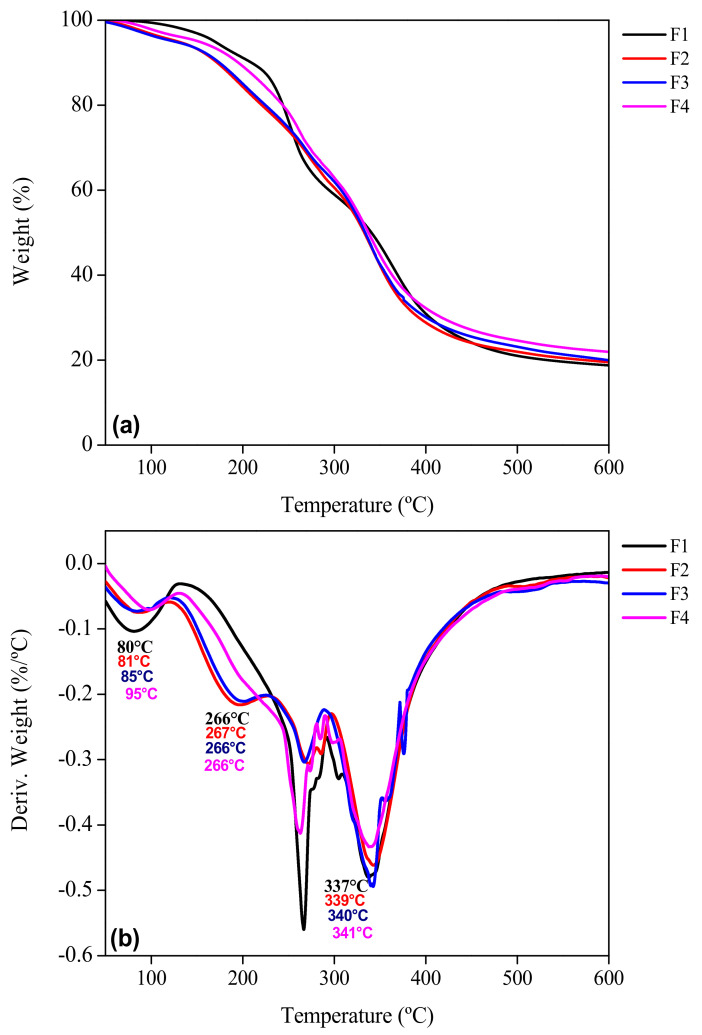
TGA profile of the reinforced gelatin biofilms. (**a**) Weight as a function of temperature and (**b**) first derivative of the weight curve. F1: control film; F2–F4: biofilm with microfiber.

**Figure 9:**
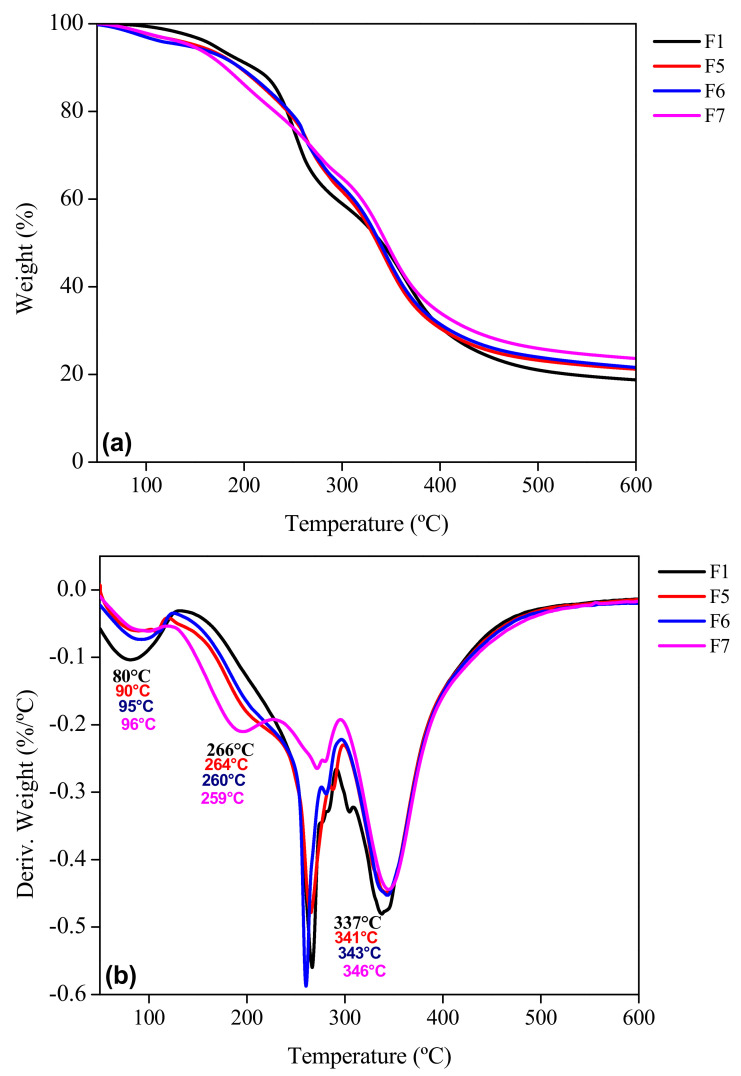
TGA profile of the reinforced gelatin biofilms. (**a**) Weight as a function of temperature and (**b**) first derivative of the weight curve. F1: control biofilm; F5–F7: biofilm with bentonite.

**Figure 10:**
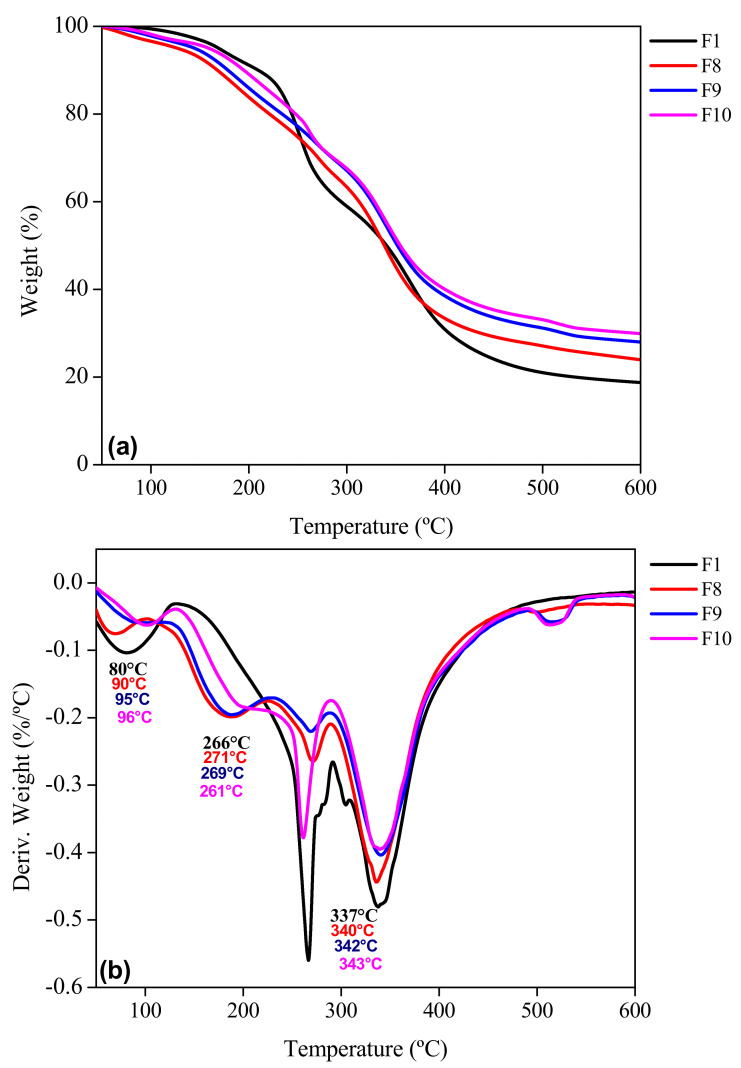
TGA profile of the reinforced gelatin biofilms. (**a**) Weight as a function of temperature and (**b**) first derivative of the weight curve. F1: control biofilm; F8–F10: biofilm with microfiber and bentonite.

The thermogravimetric analysis (TGA) and its first derivative (DTG) give information about the thermal stability of the biofilms over a wide temperature range (50 to 600 °C). Three stages of thermal degradation were observed for each sample (Figure 8a, Figure 9a and Figure 10a) where samples F2 to F10 were compared with the control biofilm F1. The F1 sample showed a temperature range for the first stage of thermal degradation between 50 and 197 °C with a weight loss of 19%. In addition, samples F2 to F4 presented a weight loss of 13–16% in the range of 50–210 °C. For samples F5 to F7, a weight loss of 12–17% in an interval of 50–214 °C was observed, while for samples F8 to F10, the interval of 50–224 °C generated a weight loss of 16–19%. These losses corresponded to adsorbed low molecular weight compounds, as well as the loss of free and bound water in the gelatin network and additives in the biofilm [22,28]. Garcia-Mendez et al. [42] reported thermogravimetric analysis of *A. angustifolia* Haw fibers, where the first stage of degradation was found in a range of 10 to 120 °C due to water evaporation with a weight loss of 5.8%. As expected, the changes observed in the different stages of weight loss as a function of temperature are in agreement with the profile of the first derivative of the thermogravimetric profile of the biofilm samples studied. In the second stage for F1, it was observed that 197–405 °C is mainly related to the degradation of the gelatin chains with a weight loss of 56–59%. A similar behavior was reported by Chuaynukul et al. [22] and Peña et al. [59].

Hoque et al. [37] attributed this degradation to the break-up of the protein chains (helical structure) and peptide bonds of the protein matrix of the control gelatin sample F1, in addition to the degradation of the glycerol molecules added as a plasticizer. The biofilms F2 to F4 mainly degraded in a temperature range of 200–429 °C with a weight loss 56–58%. Lower thermal decomposition was attributed to the presence of MF at different concentrations. García-Méndez et al. [42] observed a decomposition of agave fibers from 260 to 400 °C, which was related to the degradation of cellulosic substances, such as hemicellulose and cellulose.

Samples F5 to F7 degraded in a range of 209–447 °C with a weight loss between 54 and 58%, presenting also lower losses than the previous ones due to the inorganic content of BN. For the biofilms reinforced with both MF and BN, the degradation took place in a higher temperature range than all the others analyzed (214–452 °C) with a lower weight loss (~45–50%). The presence of both mentioned reinforcements produced a positive synergy effect in the samples. The third stage was observed from the end of the second stage up to the response of each sample at 600 °C. For F1 up to 600 °C, the residue (undegraded mass) was 5%; for F2 to F4, 4–5%; for F5 to F7, 6–7%; and for F8 to F10, 5–8%; with a total weight loss of 82%, 79–81%, 76–80% and 71–76%, respectively. The lower weight loss indicated a higher stability of samples. This last stage was related to pyrolysis, i.e., decomposition and degradation of lignin without oxygen [60]. Furthermore, it was attributed to the decomposition of the more thermally stable structure of glycerol. López-Angulo et al. [24] reported that increasing the concentration of nanoclays in their protein matrix (4 to 15%) allowed a decrease in the percentage of weight loss (72 to 61%).

The derivative thermogravimetric (DTG) curves of the samples (Figure 8b, Figure 9b and Figure 10b) showed a widening of the second stage for F2–F10 compared to F1. This could be related to a strengthening effect of the gelatin structure with the presence of micro and nano particles in the matrix, but also to the interactions identified with FTIR (Figure 3). The peak (maximum) temperatures of each stage were identified for each sample giving the ascending order of: F1, F2, F3, F4, F5, F6, F7, F8, F9 and F10, with the first stage (80, 81, 85, 95, 90, 95, 96, 90, 95 and 96 °C, respectively), second stage (266, 267, 266, 266, 264, 260, 259, 271, 269 and 261 °C) and the third stage (337, 339, 340, 341, 341, 343, 346, 340, 342 and 343 °C) corresponding to each sample indicated previously. The thermal degradation stability of the biofilms of the MF-BN mixture addition, improved markedly with increasing concentration compared to the control with no MF-BN addition.

## 4. Conclusions

The added reinforcements (MF and BN), either alone or together, had various effects on the different biofilms involved in this study. The micrographs showed agglomerations of the reinforcements on the biofilms’ surface. In the spectra by FTIR, different functional groups, corresponding to interactions between the various components were found. The solubility increased steadily in biofilm samples when the microfiber concentration increased. The moisture content among the biofilms was similar, but slightly different compared to the control. The film’s thicknesses were a function of the composition. The biofilms’ crystallinity displayed a profile showing the presence of its different components.

Strong intermolecular interactions between the protein matrix and the lignocellulosic microfiber were observed, because of their hydrophobic character leading to physical interactions. Reinforcements addition improved the barrier and thermal properties of the biofilms. BN was fundamental to improving the biofilms properties studied, due to the fractionation and dispersion of the silicate films in its composition, as well as to its affinity with polar compounds. However, the improvements observed were also due to the dispersion of both reinforcements (MF and BN) able to control the passage of water vapor through the protein matrix, making it a stronger system. The biofilms had improved properties compared to the control film (F1). Sample F8 showed the best values for barrier properties, while F9 was better for mechanical properties. Considering the overall properties, the gelatin biofilms obtained might have some potential use as food packaging. Nevertheless, further research is needed to determine the interactions in a system based on a gelatin matrix with MF and BN, but this work provided a good starting point for the physical property optimization of biofilms.

## Figures and Tables

**Table 1:** Biofilm formulations produced by casting.

Biofilms	(g/100 g Total) of Each Component				
	MF	BN	GL	GLY	DW
F1	0.0	0.0	70.0	30.0	94.0
F2	1.5	0.0	69.5	29.0	94.0
F3	3.5	0.0	68.5	28.0	94.0
F4	5.5	0.0	67.5	27.0	94.0
F5	0.0	1.5	69.5	29.0	94.0
F6	0.0	3.5	68.5	28.0	94.0
F7	0.0	5.5	67.5	27.0	94.0
F8	1.5	1.5	68.0	29.0	94.0
F9	3.5	3.5	65.0	28.0	94.0
F10	5.5	5.5	62.0	27.0	94.0

F1: control sample; F2–F4: samples with microfiber; F5–F7: samples with bentonite; F8–F10: samples with microfiber and bentonite. MF = Agave microfiber; BN = Bentonite; GL = Gelatin; GLY = Glycerol; DW = Water.

**Table 2:** Functional groups and possible vibration type detected by FTIR in the reinforced biofilms.

Wavenumber (cm^−1^)	Vibration Type	Characteristic Vibration Mode For:	Sample
3298	Stretching	Amide-A (N-H)/OH	F1–F10
2929	Stretching	Amide-B (N-H)	F1–F10
1635	Stretching	Amide I (C = O, C-N)	F1–F10
1550	Bending	Amide II (N-H, C-N)	F1–F10
1448	Deformation	C-H	F1–F10
1369	Bending	C-H- Cellulose	F2–F4, F8–F10
1236	Stretching	Amide III (N-H, C-N)	F1–F10
1090	Bending	C-O, C-C	F2–F4, F8–F10
1035	Bending Stretching	C-O, C-C Si-O	F2–C4, F8–F10 F5–F10
922	Deformation Bending	C-H, OH Al-Al-OH	F2–F4, F8–F10 F5–F10
850	Deformation	OH	F1–F10

F1: control sample; F2–F4: samples with microfiber; F5–F7: samples with bentonite; F8–F10: samples with microfiber and bentonite.

**Table 3:** Thickness and optical properties related to the biofilms color.

Sample	Thickness (μm)
F1	110.0 ± 2.6 ^a^
F2	100.0 ± 1.4 ^ab^
F3	100.0 ± 7.8 ^ab^
F4	80.0 ± 5.7 ^bc^
F5	90.0 ± 1.2 ^abc^
F6	80.0 ± 6.6 ^c^
F7	70.0 ± 1.6 ^cd^
F8	80.0 ± 1.6 ^c^
F9	70.0 ± 1.6 ^cd^
F10	60.0 ± 4.7 ^d^

Average value ± standard deviation; *n* = 3. Values within the same column followed by the same letters are not significantly different (*p* > 0.05, Tukey test). F1: control sample; F2–F4: samples with microfiber; F5–F7: samples with bentonite; F8–F10: samples with microfiber and bentonite.

## Data Availability

The data presented in this study are available on request from the first author.
